# Accuracy of Point Load Index and Brazilian Tensile Strength in Predicting the Uniaxial Compressive Strength of the Rocks: A Comparative Study

**DOI:** 10.3390/ma17205081

**Published:** 2024-10-18

**Authors:** Amin Jamshidi, Luís Sousa

**Affiliations:** 1 Department of Geology, Faculty of Basic Sciences, Lorestan University, Khorramabad 681151-44316, Iran; jamshidi.am@lu.ac.ir; 2 Department of Geology and Pole of Geosciences Center, University of Trás-os-Montes e Alto Douro, 5000-801 Vila Real, Portugal

**Keywords:** accuracy, Brazilian tensile strength, point load index, rock, uniaxial compressive strength

## Abstract

Uniaxial compressive strength (UCS) of rocks is one of the main parameters required in the design of geotechnical projects such as tunnels, dams, or rock slopes. According to the literature, there are a large number of predictive regression equations to evaluate the UCS from the point load index (PLI) and Brazilian tensile strength (BTS). However, the equations developed in previous studies have different accuracies in UCS prediction. A more accurate prediction of the UCS will result in a more appropriate design of the geotechnical project, and thus ensure its success during operation. In the present paper, a comparative study was conducted between the accuracy of PLI and BTS in predicting the UCS of the limestone and sandstone. Moreover, the role of porosity (n) on the accuracy of predicting the UCS from PLI and BTS was investigated. Some statistical indices were used to investigating the accuracy of predictive regression equations of UCS. The results revealed that the UCS of rocks can be predicted with a higher accuracy using BTS compared with PLI. Also, the findings showed that the n had a significant role in increasing the accuracy of PLI- and BTS-based regression equations of the UCS predictive. The predictive equations established in the present study can be used in practical applications for indirect evaluation of limestone and sandstone UCS in the site of a geotechnical project.

## 1. Introduction

In many geotechnical projects such as tunnels, concrete dams, or rock slopes, rocks act as foundations or surrounding materials. The strength characteristics of the rocks are among the key parameters in designing geotechnical projects. Ignoring or not paying enough attention to these characteristics can lead to design errors, and consequently the failure of the project. Any failure in the geotechnical project will result in financial loss or even loss of life. Therefore, an accurate evaluation of the rock strength characteristics and their incorporation as design parameters will increase the success of the geotechnical project during its serviceability.

The uniaxial compressive strength (UCS) is one of the most important and common of the strength characteristics of the rocks, which is used among the input parameters in the design of many geotechnical projects [1,2,3]. The well-known method of measuring the rock UCS is through the laboratory test in accordance with the guidelines provided in the international standards of the International Society for Rock Mechanics (ISRM) [4] and American Society for Testing and Materials (ASTM) [5]. In order to meet the requirements of the aforementioned standards, the specimens required to perform the UCS test must have specific dimensions in the geometric shapes of cylindrical cores or cubes. For some sedimentary rocks containing lamination (such as sandstone and shale), foliated metamorphic rocks (such as slate and schist) and highly weathered rocks such as granite, it is not possible to prepare specimens with the desired dimensions to perform the UCS testing.

A rough assessment of rock UCS is usually required in the preliminary stages of site investigation for a geotechnical project. Therefore, even if it is possible to prepare test specimens, in these steps it will not be necessary to carry out the UCS test. In this situation, indirect tools can be very valuable for a rapid evaluation of rock UCS without performing UCS laboratory tests. Regression equations are among the most common of the practical tools used in previous studies for the indirect evaluation of rock UCS. In regression equations, UCS can be predicted from other inherent characteristics of rocks such as mineralogical composition [6,7,8,9], density (ρ) [10,11], porosity (n) [2,12], water absorption (Wa) [13], point load index (PLI) [14,15], Brazilian tensile strength (BTS) [16,17], P-wave velocity (Vp) [18], Schmidt hammer hardness (SH) [19,20], Block Punch Index (BPI) [21,22], and slake durability index (SDI) [23,24]. Considering the similar nature of PLI and BTS parameters to UCS, which all represent the rock resistance to failure when exposed to external stresses, these strength parameters have been widely used in previous studies to predict the UCS through regression equations. Some predictive equations of UCS from PLI and BTS developed in the previous studies are presented in Table 1 and Table 2.

According to the ISRM [4], the dimension requirements of the rock specimen (i.e., size) for the PLI and BTS tests are almost the same. For example, both tests can be performed on cylindrical cores with a diameter and thickness of 54 and 27 mm, respectively (diameter to thickness ratio of 2). It is obvious that when it is possible to prepare rock specimens for PLI testing, these conditions also exist for BTS testing. Therefore, any PLI or BTS can be used to develop the correlation equations when the objective is to predict rock UCS. Table 1 and Table 2 indicate that there are correlation equations with various forms (i.e., linear, power, logarithmic) and different R^2^ values ranging from 0.45 to 0.98 between UCS with PLI and BTS. As a result, the PLI and BTS have different accuracies in predicting the UCS of the rocks. A more accurate prediction of the UCS will lead to a more appropriate design of rock-related geotechnical projects, including tunnels, concrete dams and rock slopes. There is a gap in previous research regarding the investigation of the accuracy of PLI and BTS in predicting the UCS of rock. Therefore, the aim of this paper is to conduct a comparative study on the accuracy of PLI and BTS in predicting the UCS of limestone and sandstone. The effect of porosity (n) on the accuracy of the correlation equations between UCS with PLI and BTS was also investigated.

## 2. Materials and Methods

**Figure 1:**
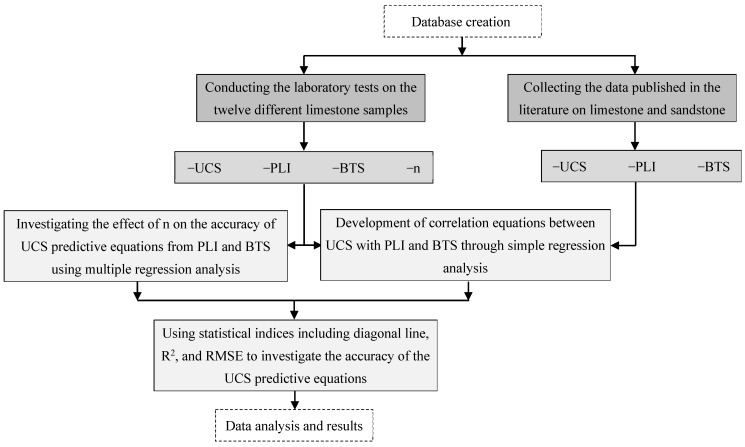
Scheme showing how the present study was carried out.

Two sets of data have been used in this paper. The first set was obtained by conducting laboratory tests and determining the UCS, PLI and BTS of twelve different limestone samples from the Lorestan province (western Iran). The other set was obtained by collecting data published in the literature on the UCS, PLI and BTS of limestones and sandstones. Correlation equations between UCS with PLI and BTS of the samples were developed by simple regression analysis. In addition, the role of n in the accuracy of UCS prediction equations based on PLI and BTS was investigated using multiple regression analysis. Some statistical indices including diagonal line (y = x), R^2^, and root mean square error (RMSE) were used to evaluate the accuracy of PLI and BTS in the UCS prediction. The flowchart of the present study is shown in Figure 1.

## 3. Database

### 3.1. Data Obtained from the Present Study

**Figure 2:**
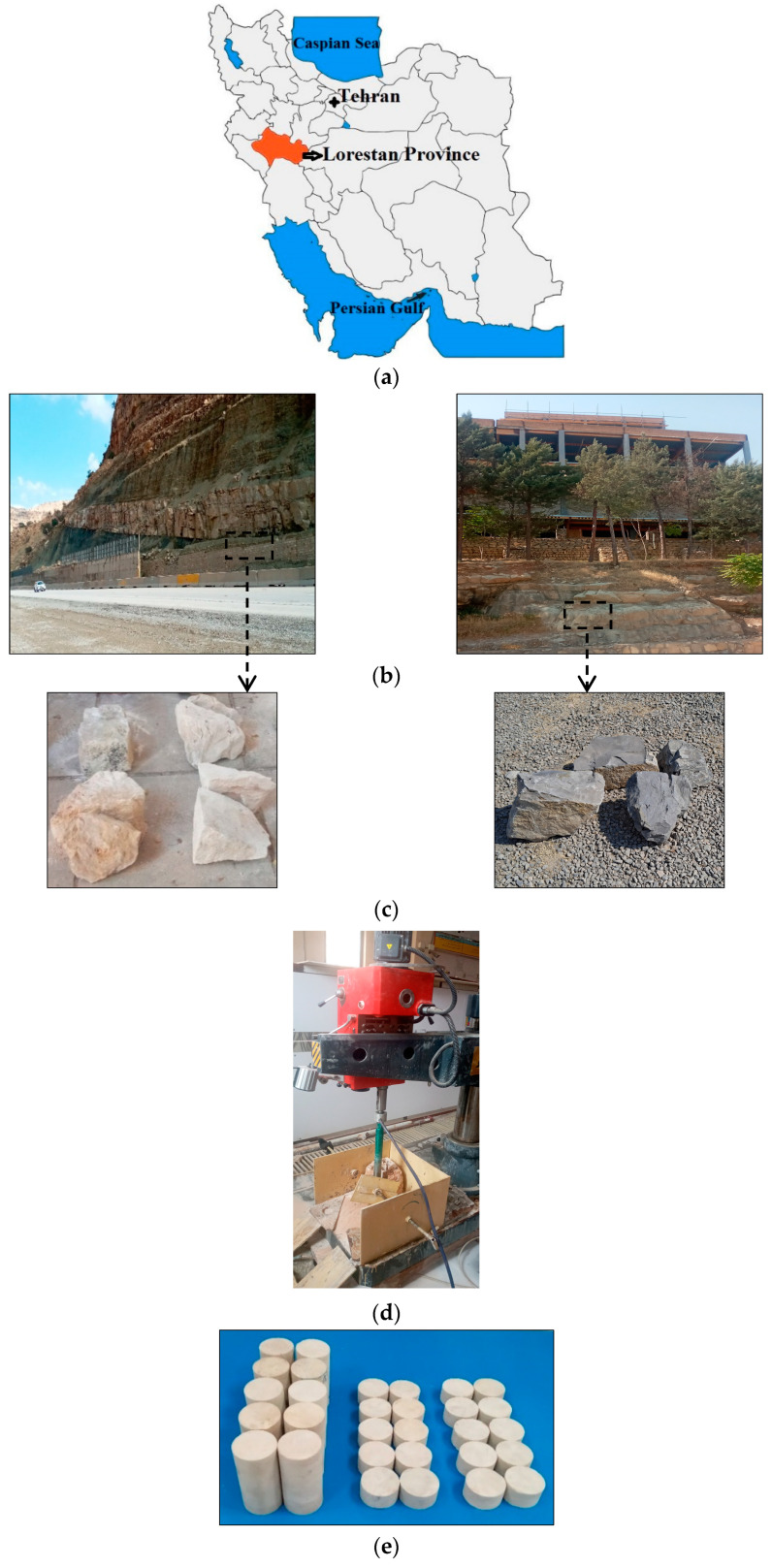
(**a**) Sampling location in Iran (Lorestan Province). (**b**) Limestone sampling locations on the rock slope of a road and the foundation of a civil structure. (**c**) Some blocks collected from limestones. (**d**) Coring machine for preparation of test specimens. (**e**) Some specimens for UCS, PLI, BTS, and n tests.

**Figure 3:**
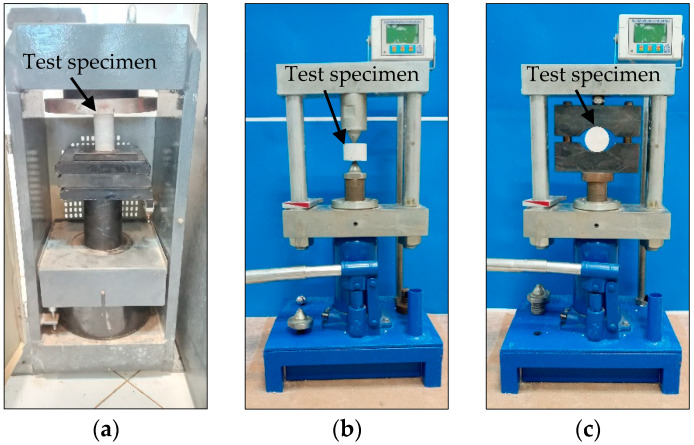
Devices used in the present study: (**a**) UCS, (**b**) PLI, and (**c**) BTS.

During several steps of field visits from some geotechnical projects in Khorramabad city (Lorestan Province, western Iran), blocks from twelve different limestone samples were collected. Figure 2 shows some of the geotechnical projects for the sampling. After transfer of block samples to the Engineering Geology Laboratory (Lorestan University), the cylindrical core specimens were prepared using a coring machine for various tests (Figure 2). The UCS, PLI, BTS, and n tests were performed on the specimens according to guidelines suggested by the International Society for Rock Mechanics [4]. Some information on the tests is presented in Table 3. Figure 3 shows the devices used in the present study to determine the UCS, PLI, and BTS of the specimens. The results are reported in Table 4. It can be seen from this table that the values of UCS, PLI, and BTS of the samples are different. These differences can be attributed to differences in photographical characteristics (i.e., mineralogical composition and textural features) and physical parameters such as density and porosity [6,7,10]. The UCS, PLI, and BTS of samples are a function of the climatic conditions prevailing in the region. Considering the climatic conditions, processes such as freezing–thawing, crystallization salt, heating–cooling, and wetting–drying can cause the deterioration of samples and, as a result, harmful effects on their UCS, PLI, and BTS [46,57].

### 3.2. Data Collected from Previous Studies

A literature review was conducted in order to collect the data required for the present study. Regarding the comparison of the accuracy of PLI and BTS in predicting the UCS of the rocks, only the papers that contained all these parameters simultaneously were applicable for the present study. For this, UCS, PLI, and BTS data published by Khajevand [18], Teymen and Menguc [56], Jamshidi et al. [59], and Lakirouhani, et al. [60] were extracted as a database for the present study. Table 5, Table 6, Table 7 and Table 8 present the UCS, PLI, and BTS data from these researchers.

## 4. Data Analysis and Results

### 4.1. Comparing the Accuracy of PLI and BTS in Predicting the UCS

The data presented in Table 4, Table 5, Table 6, Table 7 and Table 8 were used to develop UCS predictive equations from PLI and BTS. For this, simple regression analyses were performed on the data. The four types of regression curves including linear (y = ax + b), power (y = ax^b^), exponential (y = ae^x^), and logarithmic (y = a + ln x) between UCS with PLI and BTS were fitted. The R^2^ value was used as a comparative measure to select the most appropriate regression curve. Among the regression curves, the one with the highest R^2^ was chosen as the correlation equation between UCS with PLI and BTS.

**Figure 4:**
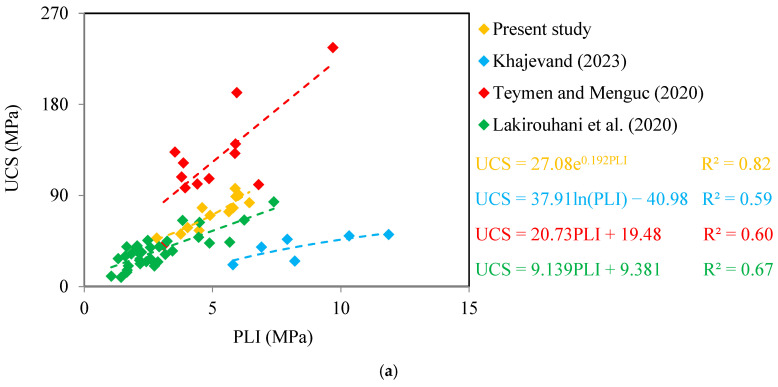

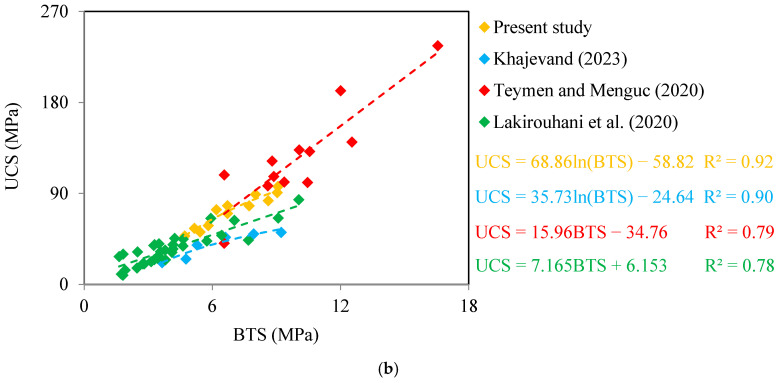
Predictive equations of UCS for limestone using (**a**) PLI and (**b**) BTS (Khajevand [18], Teymen and Menguc [56], Lakirouhani et al. [60]).

**Figure 5:**
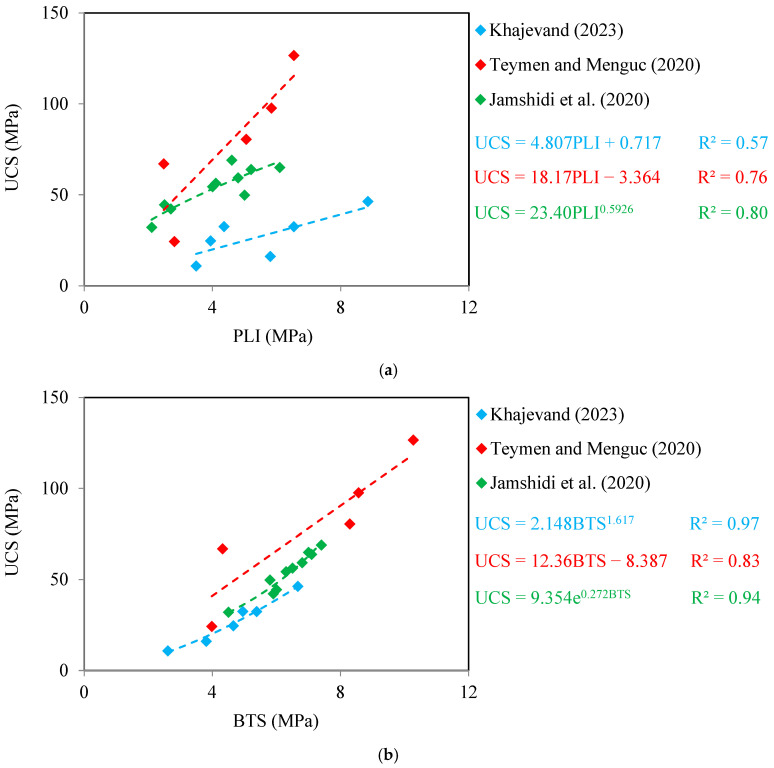
Predictive equations of UCS for sandstone using (**a**) PLI and (**b**) BTS (Khajevand [18], Teymen and Menguc [56], Jamshidi et al. [59]).

**Figure 6:**
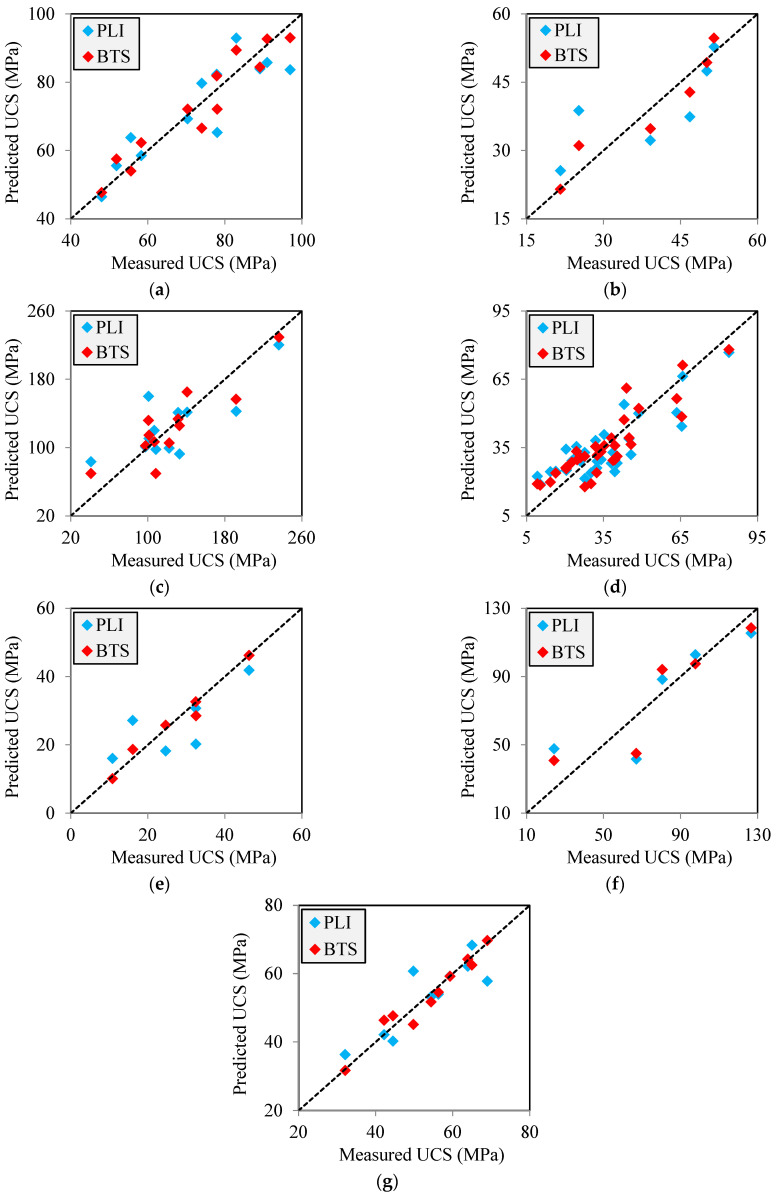
Measured UCS versus predicted UCS using the PLI and BTS: (**a**) present study (limestone), (**b**) Khajevand [18] (limestone), (**c**) Teymen and Menguc [56] (limestone), (**d**) Lakirouhani et al. [60] (limestone), (**e**) Khajevand [18] (sandstone), (**f**) Teymen and Menguc [56] (sandstone), and (**g**) Jamshidi et al. [59] (sandstone).

Figure 4 and Figure 5 show correlation equations between UCS with PLI and BTS for limestone and sandstone, respectively. It can be seen from these figures that the UCS of the samples shows an increasing trend with the increase of PLI and BTS. These results are in good agreement with the findings of Sadeghi et al. [2], Rajabzadeh et al. [12], Liu et al. [61], and Garrido et al. [62] on limestones. There are correlation equations in the forms of linear, power, exponential, and logarithmic equations between UCS with PLI and BTS with R^2^ values from 0.60 to 0.97. These values are acceptable, showing meaningful correlations between UCS with PLI and BTS. The PLI-based correlation equations have different R^2^ values than the BTS-based correlation equations, indicating that the PLI and BTS have different accuracies in predicting the UCS of the samples. The diagonal line (y = x) is a common qualitative measure and widely used by researchers to compare the accuracy between two correlation equations to predict an unknown parameter of rock [3,37,57]. The measured UCS values of the samples (Table 4, Table 5, Table 6, Table 7 and Table 8) were plotted versus predicted UCS using correlation equations shown in Figure 4 and Figure 5. The results of these analyses are shown in Figure 6. A data point on the diagonal line indicates an exact prediction of UCS using the PLI- or BTS-based correlation equations, i.e., the predicted UCS is in agreement with the measured UCS. Increasing the distance of the data points from the diagonal line increases the prediction error of UCS using the correlation equation. It can be seen from Figure 6 that for all databases, the predicted UCS values using the BTS are more concentrated around the diagonal line than those obtained using the PLI. The comparison of the scatter of the data points around the diagonal line indicates that the BTS is a more accurate parameter to predict the UCS of the samples.

As two quantitative indices, the R^2^ and RMSE were used for a deeper investigation of the accuracy of UCS predictive equations developed based on PLI and BTS. These two indices are among the most common and frequently used statistical parameters to assess the accuracy of a correlation equation [2,58,63]. The degree of fitting data to a regression curve can be measured by the R^2^ value, which measures the proportion of variation in the dependent variable. The RMSE, on the other hand, measures the average difference between the predicted values and the measured values. Mathematically, it is the standard deviation of the residuals. Residuals represent the distance between the regression curve and the data points. It should be mentioned that a correlation equation with a higher R^2^ has a smaller RMSE. In general, between two correlation equations, the one with the higher R^2^ and the smaller RMSE is more accurate in predicting an unknown parameter of rock. The R^2^ and RMSE values of correlation equations established between the UCS with PLI and BTS (Figure 4 and Figure 5) were calculated by Equations (1) and (2), respectively:

(1) $$R^{2}=\frac{\sum_{i=1}^{N}\left(y-\bar{y}\right)\left(y^{’}-{\bar{y}}^{’}\right)}{\sqrt{\sum_{i=1}^{N}{\left(y-\bar{y}\right)}^{2}{\left(y^{’}-{\bar{y}}^{’}\right)}^{2}}}$$

(2) $$RMSE=\sqrt{\frac{1}{N}\sum_{i=1}^{N}{\left(y-y^{’}\right)}^{2}}$$

where y and y’ are the measured and predicted values of the UCS, respectively, ȳ and ȳ’ are mean values of the y and y’, respectively, and N is the number of the dataset.

**Figure 7:**
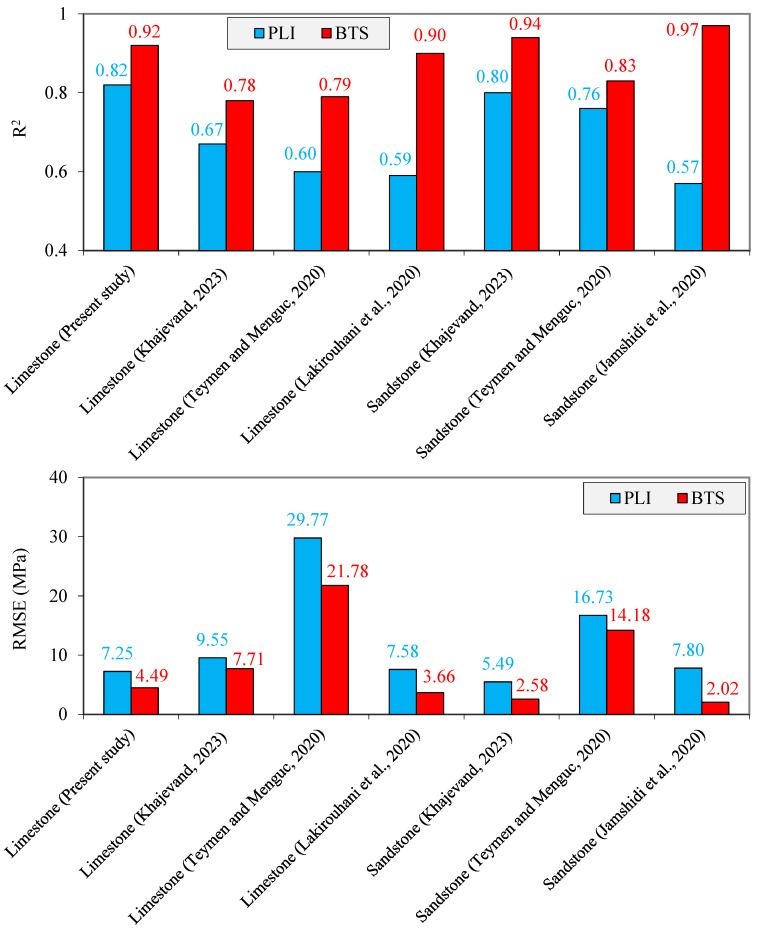
The R^2^ and RMSE values of the correlation equations between UCS with PLI and BTS (Khajevand [18], Teymen and Menguc [56], Jamshidi et al. [59], Lakirouhani et al. [60]).

The R^2^ and RMSE values are presented in Figure 7. According to this figure, the correlation equations between UCS and PLI have values of R^2^ from 0.60 to 0.82, while the correlation equations between UCS and BTS show a higher R^2^, ranging from 0.78 to 0.97. The UCS predictive equations based on PLI and BTS have RMSE values between 5.49–29.77 MPa and 2.02–21.78 MPa, respectively. In regression analyses, R^2^ and RMSE values can range from 0 to 1 and 0 to positive infinity, respectively. The values of 1 and 0 values for the R^2^ and RMSE, respectively, obtained from a correlation equation means that the predicted values are in perfect agreement with the measured values. A correlation equation with the high R^2^ and a low RMSE indicates that the model fits the data well and has more accurate predictions. Conversely, lower R^2^ and higher RMSE values suggest more errors and a less accurate predictive equation. Comparing the values of R^2^ and RMSE of the correlation equations reveals that BTS, compared to PLI, is more accurate in predicting the rocks UCS. These results are in good agreement with the findings obtained from diagonal lines developed between the predicted and measured UCS values (Figure 6).

**Figure 8:**
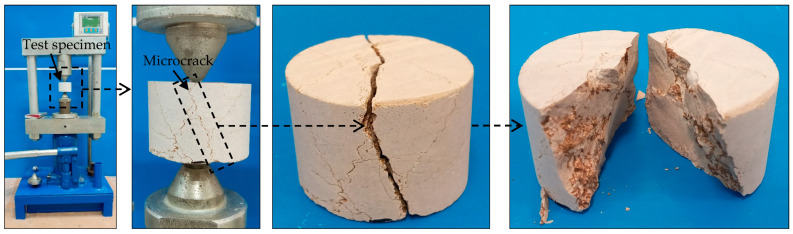
Heterogeneity in the rock specimen and its premature failure during the PLI test.

According to the International Society for Rock Mechanics [4], the shape and size of the rock specimen for PLI and BTS tests are similar, i.e., both were cylindrical cores with a diameter–thickness ratio of 2 (diameter = 54 mm). The findings showed that the BTS is a more accurate parameter than the PLI in predicting the UCS of the rocks. This can be attributed to the nature of the PLI device and its loading system on the rock specimen. In the PLI test, the loading on the specimen was carried out through two conical pieces with an apex angle of 60° (Figure 8). Therefore, the load applied on the rock specimen will be point. Rocks have a heterogeneous nature due to the variety in mineralogical composition, and the presence of pores and microcracks [64,65]. When the rock specimen is subjected to the PLI test, a pore or microcrack may be present along the axis of the loading between the conical pieces. This situation is shown schematically in Figure 8. The pores or microcrack will cause the specimen to failure at a lower load value, and therefore a lower PLI value compared to the actual PLI value. Figure 8 presents the premature failure of a specimen containing microcracks (Limestone 9 in the present study). According to this figure, it can be seen that the rupture surface of the specimen has passed across a microcrack. This will result in measurement error in the PLI, and therefore a prediction error of the UCS using the PLI-based correlation equation.

### 4.2. Effect of n on the Accuracy of UCS Predictive Equations

Multiple regression analyses were applied to the data to investigate the effect of n on the accuracy of UCS prediction from PLI and BTS. In these analyses, the UCS was regarded as the dependent variable, and PLI, BTS, and n were considered as independent variables. The general forms of the multiple correlation equations are as follows:

(3) $$UCS=\beta_{0}+\beta_{1}PLI-\beta_{2}n$$

(4) $$UCS=\beta_{0}+\beta_{1}BTS-\beta_{2}n$$

where UCS is the predicted value of the uniaxial compressive strength, PLI, BTS, and n are point load index, Brazilian tensile strength, and porosity, respectively, β_0_ is a constant, and β_1_ and β_2_ are the regression coefficients.

The data presented in Table 4 were analyzed using the SPSS^®^v.19 code statistical software. Multiple regression analyses were undertaken with the significance level of 5% and the best-fit curves were obtained between variables using the least squares method. The results of multiple regression analyses are given in Table 9 (Equations (6) and (8)). The significance and global usefulness of the multiple correlation equations were checked using the variance analysis for the regressions. F statistics test is frequently used in regression and variance analysis. The results of variance analysis for the multiple correlation equations are given in Table 9. For a significance level of 5%, the value of tabulated F with the degree of freedom υ_1_ = 2 and υ_2_ = 9 is 4.26. Since the F values computed for the multiple correlation equations are larger than the value of tabulated F, it can be concluded that the multiple correlation equations have good validity to predict UCS from PLI, BTS, and n.

The R^2^ and RMSE values of the simple and multiple correlation equations were used to investigate the role of n on the accuracy of UCS prediction. According to results given in Table 9, R^2^ and RMSE values equal to 0.82 and 7.25, respectively, were obtained for the simple correlation equation between UCS and PLI (Equation (5)), while these values were 0.92 and 4.69 for the multiple correlation equation between UCS with PLI and n (Equation (6)). The simple correlation equation between UCS and BTS (Equation (7)) had R^2^ and RMSE of 0.92 and 4.28, respectively. Also, R^2^ and RMSE values for multiple correlation equation between UCS with BTS and n (Equation (8)) were equal to 0.94 and 3.96, respectively. The comparison of these values shows that the multiple correlation equations rather than the simple ones have higher R^2^ and lower RMSE values, indicating that the n increases the accuracy of UCS prediction from PLI and BTS. As a result, prediction accuracy of the multiple correlation equations is higher than those of simple correlation equations. This is a significant finding regarding the more accurate evaluation of rocks UCS in the preliminary steps of the site investigation of a geotechnical project such as a tunnel, concrete dam, or rock slope. However, the correlation equations developed in the present study (Equations (5)–(8)) should be used with caution for limestones from other regions with a range of values for UCS, PLI, BTS and n similar to the samples tested in the present study (Table 4). This may be accompanied by some prediction errors.

Although our results revealed that multiple correlation equations compared to simple ones have higher accuracy in predicting the rocks UCS, a literature review was also conducted in this regard. Some researchers such as Gokceoglu and Zorlu [44], Teymen and Menguc [56], Mishra and Basu [66], Azimian [67], and Farhadian et al. [68] showed that compared with simple correlation equations, using the multiple correlation equations can provide a predictive model of UCS with a higher R^2^ value, and thus a more accurate prediction. However, the findings of Sadeghi et al. [2], Rajabzadeh et al. [12], Kamani and Ajalloeian [69], and Diamantis et al. [70] differ from those obtained by the above-mentioned researchers. Based on the regression analyses on the UCS, BTS, PLI, and n of the limestones, these researchers concluded that there is no significant difference between the accuracy of simple and multiple correlation equations in predicting the UCS.

**Figure 9:**
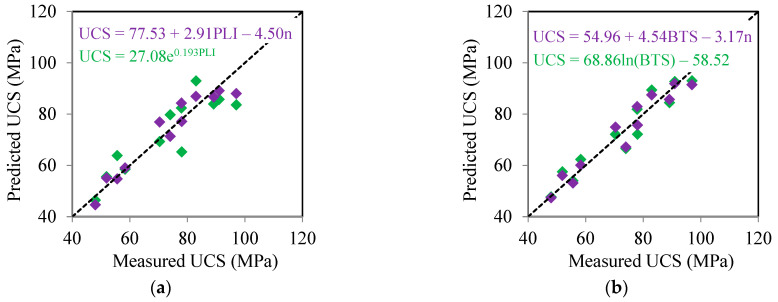
Measured UCS versus predicted UCS using simple and multiple correlation equations: (**a**) PLI (Equations (5) and (6)), and (**b**) BTS (Equations (7) and (8)).

In addition to the quantitative criteria of R^2^ and RMSE, a qualitative comparison was also carried out to evaluate the effect of n on the accuracy of the UCS predictor equations. For this, the measured UCS values were plotted versus predicted UCS values obtained from the simple and multiple correlation equations. As can be seen from Figure 9, the data points obtained based on the multiple correlation equations are closer to the diagonal line, which reveals that these equations compared to the simple correlation equations have higher accuracy for prediction of rocks UCS under study. These findings are in line with results obtained from R^2^ and RMSE values of the simple and multiple correlation equations. Based on Figure 9, a valuable point is that the data points are closer to the diagonal line for the BTS-based multiple correlation equation (Equation (8)) compared to the PLI-based ones (Equation (6)). Consequently, the BTS-based multiple correlation equation shows that it has a higher accuracy for predicting the UCS of rocks.

## 5. Conclusions

Based on laboratory tests in the present study and a literature review of previous studies, a database including uniaxial compressive strength (UCS), point load index (PLI), Brazilian tensile strength (BTS), and porosity (n) of different limestones and sandstones was created. Using data analyses, two goals were pursued: (1) investigating the accuracy of PLI and BTS in predicting UCS, and (2) the role of n in the prediction accuracy of UCS using of PLI and BTS-based correlation equations. The main findings of the present study are as follows:

- The results of simple regression analyses showed that the BTS provides a more accurate prediction of the rock UCS than the PLI. This was verified by comparing the statistical indices (including diagonal line (y = x), coefficient of determination (R^2^), and root mean square error (RMSE)) obtained for PLI and BTS-based correlation equations.
- The lower accuracy of PLI compared to the BTS in the indirect assessment of UCS is due to nature of the PLI device and its loading system on the rock specimen. The results showed that the specimen heterogeneity (caused by the presence of pores or microcracks) strongly affects the accuracy of PLI measurements and thus the performance of the UCS prediction equation.
- Based on the comparison of the diagonal line results and the R2 and RMSE values obtained from the simple and multiple regression analyses, the n has a significant effect on the predictive accuracy of the UCS from the PLI and BTS-based correlation equations.
- Considering the same size of the rock specimen for PLI and BTS tests, it is recommended that BTS measurements be preferred. As a result, a more accurate evaluation of the rock UCS can be obtained in the preliminary stages of the site investigation of a geotechnical project such as a tunnel, concrete dam or rock slope. A more accurate evaluation of the UCS will lead to a more appropriate design of the geotechnical project, thereby increasing its long-term success.
- As an important point, it is necessary for researchers to carry out more studies in the future to investigate the accuracy of PLI and BTS for other types of rock.

## Figures and Tables

**Table 1:** Prediction of the UCS from PLI.

Reference	Rock Type	Predictive Equation	R^2^
Ulusay et al. [6]	Sandstone	UCS = 19PLI + 12.7	0.81
Akbay [15]	Limestone, Marble	UCS = 13.71PLI + 5.51	0.71
Singh and Singh [25]	Quartzite	UCS = 23.37PLI	0.96
Tugrul and Zarif [26]	Granitic rocks	UCS = 15.25PLI	0.96
Lashkaripour [27]	Mudrock	UCS = 21.4PLI	0.85
Tsiambaos and Sabatakakis [28]	Different rock types	UCS = 7.3PLI^1.71^	0.82
Zorlu et al. [29]	Sandstone	UCS = 10.3PLI + 28.1	0.76
Fener et al. [30]	Different rock types	UCS = 9.08PLI + 39.3	0.72
Kahraman et al. [31]	Different rock types	UCS = 10.9PLI + 27.4	0.61
Basu and Aydin [32]	Granite	UCS = 21PLI	0.93
Yilmaz and Yuksek [33]	Gypsum	UCS = 10.5PLI − 3.97	0.57
Mishra and Basu [34]	Sandstone	UCS = 13.0PLI − 5.19	0.84
Singh et al. [35]	Limestone	UCS = 22.3PLI	0.68
Palassi and Emami [36]	Travertine, Marble	UCS = 20.1PLI − 17.1	0.80
Azimian and Ajalloeian [37]	Marl	UCS = 56.94 ln(PLI) − 1.66	0.93
Yin et al. [38]	Granitic rocks	UCS = 22.27PLI	0.82
Sadeghiamirshahidi and Vitton [39]	Gypsum	UCS = 6.58PLI	0.91
Rabat et al. [40]	Siltstone	UCS = 14.26PLI	0.98
Jamshidi [41]	Sandstone	UCS = 4.94PLI + 33.03	0.85
Kong et al. [42]	Different rock types	UCS = 16.19PLI	0.90

**Table 2:** Prediction of the UCS from BTS.

Reference	Rock Type	Predictive Equation	R^2^
Sadeghi et al. [2]	Carbonate rocks	UCS = 7.26BTS	0.95
Karman et al. [16]	Different rock types	UCS = 4.87BTS + 24.30	0.90
Iyare et al. [17]	Argillite	UCS = 5.31BTS^1.06^	0.87
Tugrul and Zarif [26]	Granite, Granodiorite	UCS = 6.67BTS + 0.73	0.92
Bell and Lindsay [43]	Sandstone	UCS = 6.71BTS + 36.0	0.61
Gokceoglu and Zorlu [44]	Graywacke	UCS = 6.8BTS + 13.5	0.65
Altindag and Guney [45]	Limestone, Granite, Marble	UCS = 2.38BTS^1.073^	0.79
Farah [46]	Sedimentary rocks	UCS = 7.86BTS − 447.63	0.92
Tahir et al. [47]	Sedimentary rocks	UCS = 7.53BTS	0.45
Kahraman et al. [48]	Different rock types	UCS = 10.61BTS	0.54
Basu et al. [49]	Sandstone	UCS = 10.53BTS − 10.23	0.83
Yesiloglu-Gultekin et al. [50]	Granite, Granodiorite	UCS = 7.22BTS + 40.08	0.61
Kallu and Roghanchi [51]	Igneous rocks	UCS = 6.75BTS^1.08^	0.80
Fereidooni [52]	Hornfels schist	UCS = 10.03BTS + 55.19	0.92
Ribeiro et al. [53]	Sedimentary rocks	UCS = 13.70BTS	0.67
Masoumi et al. [54]	Sandstone	UCS = 9.29BTS + 3.91	0.68
Aliyu et al. [55]	Flint	UCS = 10.4BTS + 18.2	0.63
Teymen and Menguc [56]	Andesite, Limestone, Marble	UCS = 7.73BTS^1.197^	0.90
Arman [57]	Gypsum	UCS = 4.233BTS + 13.64	0.53
Khajevand [58]	Limestone	UCS = 40.09ln(BTS) − 36.14	0.94

**Table 3:** Information about the specimens used for UCS, PLI, BTS, and n tests.

Test	Specimen Shape	Specimen Size			Specimen Status	Specimen Number	Source
		Diameter (mm)	Length (mm)	D to L			
UCS	Cylindrical core	44	88	2.0	Dry	5	ISRM [4]
PLI	Cylindrical core	44	30	~1.5	Dry	5	ISRM [4]
BTS	Cylindrical core	44	30	~1.5	Dry	5	ISRM [4]
n	Cylindrical core	44	30	~1.5	Dry	5	ISRM [4]

**Table 4:** The UCS, PLI, BTS, and n of limestone samples obtained in the present study.

Sample Code	UCS (MPa)	PLI (MPa)	BTS (MPa)	n (%)
Limestone 1	78.04 (3.72) ^1^	4.59 (0.39)	6.70 (0.29)	3.05 (0.20)
Limestone 2	51.90 (3.24)	3.75 (0.38)	5.42 (0.25)	7.41 (0.17)
Limestone 3	74.00 (2.71)	5.63 (0.36)	6.18 (0.20)	5.01 (0.19)
Limestone 4	58.33 (4.35)	4.02 (0.43)	5.81 (0.31)	6.71 (0.24)
Limestone 5	77.90 (2.50)	5.80 (0.37)	7.72 (0.22)	2.24 (0.18)
Limestone 6	48.00 (3.48)	2.82 (0.38)	4.70 (0.26)	9.12 (0.21)
Limestone 7	89.12 (4.70)	5.90 (0.45)	8.01 (0.34)	1.78 (0.23)
Limestone 8	55.60 (5.11)	4.47 (0.48)	5.15 (0.35)	7.94 (0.19)
Limestone 9	91.00 (3.81)	6.01 (0.41)	9.03 (0.32)	1.30 (0.25)
Limestone 10	96.97 (2.98)	5.88 (0.37)	9.07 (0.24)	1.47 (0.13)
Limestone 11	70.35 (3.77)	4.90 (0.39)	6.70 (0.26)	3.30 (0.24)
Limestone 12	82.96 (4.02)	6.43 (0.40)	8.61 (0.29)	2.07 (0.17)

^1^ The value in the bracket is standard deviation.

**Table 5:** The UCS, PLI, and BTS in the study of Khajevand [18].

Rock Type	UCS (MPa)	PLI (MPa)	BTS (MPa)
Limestone	50.13	10.32	50.13
Limestone	25.19	8.20	25.19
Limestone	51.51	11.86	51.51
Limestone	39.12	6.90	39.12
Limestone	21.60	5.79	21.60
Limestone	46.82	7.91	46.82
Sandstone	46.29	8.85	6.67
Sandstone	32.46	6.54	5.38
Sandstone	16.09	5.80	3.81
Sandstone	32.51	4.36	4.95
Sandstone	24.65	3.94	4.65
Sandstone	10.82	3.49	2.61

**Table 6:** The UCS, PLI, and BTS in the study of Teymen and Menguc [56].

Rock Type	UCS (MPa)	PLI (MPa)	BTS (MPa)
Limestone	140.92	5.89	12.53
Limestone	236.19	9.69	16.55
Limestone	191.74	5.94	12.00
Limestone	97.62	3.93	8.59
Limestone	40.89	3.09	6.55
Limestone	108.40	3.79	6.55
Limestone	133.00	3.53	10.06
Limestone	100.80	6.79	10.45
Limestone	101.35	4.40	9.36
Limestone	131.58	5.87	10.55
Limestone	122.20	3.86	8.79
Limestone	106.75	4.86	8.87
Sandstone	97.64	5.84	8.56
Sandstone	24.29	2.81	3.98
Sandstone	80.48	5.05	8.29
Sandstone	66.98	2.48	4.32
Sandstone	126.60	6.54	10.27

**Table 7:** The UCS, PLI, and BTS in the study of Jamshidi et al. [59].

Rock Type	UCS (MPa)	PLI (MPa)	BTS (MPa)
Sandstone	54.4	4.0	6.3
Sandstone	65.0	6.1	7.0
Sandstone	63.9	5.2	7.1
Sandstone	42.2	2.7	5.9
Sandstone	56.3	4.1	6.5
Sandstone	44.5	2.5	6.0
Sandstone	69.0	4.6	7.4
Sandstone	49.8	5.0	5.8
Sandstone	32.1	2.1	4.5
Sandstone	59.3	4.8	6.8

**Table 8:** The UCS, PLI, and BTS in the study of Lakirouhani et al. [60].

Rock Type	UCS (MPa)	PLI (MPa)	BTS (MPa)
Limestone	31.84	3.15	31.84
Limestone	27.62	2.57	27.62
Limestone	24.69	2.41	24.69
Limestone	22.65	2.17	22.65
Limestone	20.37	2.73	20.37
Limestone	16.38	1.67	16.38
Limestone	24.47	2.86	24.47
Limestone	25.97	2.15	25.97
Limestone	20.48	1.71	20.48
Limestone	14.26	1.65	14.26
Limestone	9.18	1.43	9.18
Limestone	10.30	1.05	10.30
Limestone	43.93	5.66	43.93
Limestone	32.31	1.83	32.31
Limestone	39.37	2.91	39.37
Limestone	30.10	1.59	30.10
Limestone	34.01	2.23	34.01
Limestone	32.52	2.15	32.52
Limestone	35.22	3.43	35.22
Limestone	27.67	1.32	27.67
Limestone	43.00	4.88	43.00
Limestone	44.93	3.23	44.93
Limestone	63.51	4.49	63.51
Limestone	48.70	4.45	48.70
Limestone	45.66	2.47	45.66
Limestone	39.38	1.65	39.38
Limestone	40.31	2.06	40.31
Limestone	38.65	2.57	38.65
Limestone	38.06	2.05	38.06
Limestone	83.81	7.38	83.81
Limestone	65.82	6.23	65.82
Limestone	65.45	3.83	65.45

**Table 9:** The statistical test results of the simple and multiple correlation equations developed in the present study (data from Table 4).

Equation No.	Equation Type	R^2^	RMSE (MPa)	F Value		F Sig.
				Computed	Tabulated	
(5)	UCS = 27.08e^0.193PLI^	0.82	7.25	-	-	0.000
(6)	UCS = 77.53 + 2.91PLI − 4.50n	0.92	4.28	54.96	4.26	0.000
(7)	UCS = 68.86ln(BTS) − 58.82	0.92	4.49	-	-	0.000
(8)	UCS = 54.96 + 4.54BTS − 3.17n	0.94	3.96	64.84	4.26	0.000

## Data Availability

The original contributions presented in the study are included in the article, further inquiries can be directed to the corresponding author.
